# Guillain-Barré Syndrome in the COVID-19 Pandemic

**DOI:** 10.3390/neurolint14010003

**Published:** 2021-12-24

**Authors:** Abdullah Ahmad Tawakul, Amal Waleed Al-Doboke, Shahad Ali Altayyar, Seham Abdulhafith Alsulami, Ahlam Musallam Alfahmi, Raghad Turki Nooh

**Affiliations:** 1 Faculty of Medicine, Department of Medicine, Umm Al Qura University, Al-Abdia Main Campus, Makkah 21955, Saudi Arabia; 2 College of Medicine, Umm Al-Qura University, Al-Abdia Main Campus, Makkah 21955, Saudi Arabia; amal.doboki1419@gmail.com (A.W.A.-D.); shahdaltayyar@gmail.com (S.A.A.); sehamabdulhafith@gmail.com (S.A.A.); ahlamalfhmi754@gmail.com (A.M.A.); Ra.turki@gmail.com (R.T.N.)

**Keywords:** Guillain–Barré syndrome, GBS, SARS-CoV-2, COVID-19, SARS

## Abstract

There have been several reported cases of severe acute respiratory syndrome (SARS-CoV-2) infection that were associated with an increased incidence of neurological manifestations, including Guillain–Barré syndrome (GBS). This review aims to present information on the reports of GBS associated with coronavirus disease 2019 (COVID-19) infection. Our review is retrospective work examining articles published from the 1 April 2020 to the 8 May 2021 in the English language. We used the diagnostic criteria and classification published by the National Institute of Neurological Disorders and Stroke and Brighton Collaboration. GBS is usually a postinfectious syndrome, but GBS in the COVID-19 pandemic also takes on a para-infectious profile. In the reports, the genetic factor has a role in developing GBS in some patients. In conclusion, the association between COVID-19 and GBS is not very clear. Still, one mechanism is strongly associated with COVID-19 and immune-mediated neurological complications, which is molecular mimicry between SARS-CoV-2 and human autoantigens.

## 1. Introduction

Over the past two decades, coronaviruses have caused three pandemic infections, known as severe acute respiratory syndrome (SARS), the middle east respiratory syndrome (MERS), and coronavirus disease 2019 (COVID-19). Each of these three infections is caused by coronaviruses belonging to the beta genus. Infections caused by these beta coronaviruses show various clinical symptoms, from asymptomatic to severe illness and mortality. The first pandemic was reported in 2002–2003 in Guangdong, China, due to severe acute respiratory syndrome coronavirus 1 (SARS-CoV-1), and middle east respiratory syndrome coronavirus (MERS-CoV) caused the second pandemic. It was first detected in the Kingdom of Saudi Arabia in 2012. Severe acute respiratory syndrome coronavirus 2 (SARS-CoV-2) is a member of the Coronaviridae subfamily and causes the COVID-19 pandemic [1].

The present-day pandemic resulted from a novel coronavirus named SARS-CoV-2 and was first identified in December 2019 in Wuhan in China. On 30 January 2020, the World Health Organization declared the SARS-CoV-2 an international public health emergency [2]. Up to now, millions of cases have been confirmed worldwide, and the USA has been one of the most affected countries [3]. 

SARS-CoV-2 is the most recent novel coronavirus to emerge and has spread globally in the last two years. At the beginning of the pandemic, it was thought that bats were the natural reservoir for SARS-CoV-2, but nowadays, it is suggested that individuals are infected via an intermediate host, such as a pangolin. The most common mode of transmission is thought to be from a droplet that is expelled during face-to-face exposure, either while talking, coughing, or sneezing. Another possible mode of transmission is through contact with surfaces. The viral load reaches the peak in the upper respiratory tract, usually when symptoms start to appear, but viral shedding begins about two to three days before symptoms become onset. According to this fact, the asymptomatic and presymptomatic carrier can transmit the virus to others at this phase. It is thought that the presymptomatic transmission of the virus is a significant cause of the spread of SARS-CoV-2, because asymptomatic or presymptomatic individuals do not yet know that they are carriers until the symptoms appear; by this time, they may infect other individuals. SARS-CoV-2, when it enters the individual’s body, targets certain cells, such as nasal, bronchial epithelium, and pneumocyte cells via the viral spike proteins, which bind to angiotensin-converting enzyme 2 (ACE2) receptors. In another site, the host cells have an enzyme called type 2 transmembrane serine protease (TMPRSS2), which enhances the viral uptake by the cleavage of ACE2 and activating the SARS-CoV-2 protein. SARS-CoV-2 is similar to any other respiratory viral disease, in that it can cause lymphopenia by infecting and killing the T-lymphocyte, and it impairs lymphopoiesis, inducing lymphocyte apoptosis. Besides cell-mediated immunity, it also disrupts humoral immunity. Furthermore, SARS-CoV-2 infects the pulmonary capillary endothelial cells, worsening the inflammatory response and increasing the infiltration of the monocytes and neutrophils. In the last stage of the infection, the epithelial endothelial barrier’s integrity will be compromised [4].

Recently, numerous case reports have indicated an association between the incidence of Guillain–Barré syndrome (GBS) and previous SARS-CoV-2 infection, which preceded GBS onset by up to four weeks. Therefore, a postinfectious dysregulation of the immune system, caused by SARS-CoV2, was found to be the most probable trigger [5]. 

COVID-19 is a systemic disorder that typically presents with fever and respiratory symptoms. Recently, the symptoms of COVID-19 were found to be dependent on age, the patient’s underlying medical conditions, and the condition of the immune system. There are increasing case reports of GBS in SARS-CoV-2 infection, which may suggest a possible association between GBS and COVID-19 [1,2,6].

SARS-CoV-2 infection may be associated with an increased incidence of neurological manifestations; studies from the current pandemic have reported COVID-19 patients presenting with dizziness, headache, myalgias, hypogeusia, and hyposmia. There were more severe symptoms involving GBS, myositis, cerebrovascular diseases, encephalitis, and encephalopathy. The clinical scale and consequences of neurologic manifestations related to SARS-CoV-2 infection were widely varied, indicating various underlying pathogenic processes [7,8].

One of the neurological complications of COVID-19 infection is GBS. GBS is an autoimmune neurological disorder that affects the peripheral nervous system. GBS manifests as progressive weakness of the limbs and loss or reduction in reflexes; the diagnosis of GBS depends on the results of clinical, electrophysiological, and cerebrospinal fluid (CSF) examinations [9,10]. In this disorder, protein concentrations in the CSF increase, while the white cell count is average. A viral or bacterial infection commonly triggers GBS. In response to the antigen, the immune system is stimulated, and the nerve roots and peripheral nerves are damaged due to the structural similarity of this antigen to axons and myelin. The symptoms peak within four weeks, and the patients should be observed because 20 to 30% of them will require mechanical ventilation [9].

This review aims to present information on the reports of GBS associated with the COVID-19 infection. This review discusses the association between GBS and COVID-19, diagnostic criteria, clinical features, laboratory and imaging, management, complications, and death related to COVID-19 infection with concomitant GBS. In addition to that, we discuss the overlap between COVID-19 pneumonia and the respiratory muscle dysfunction associated with GBS. 

## 2. Methods

This review is a retrospective work examining articles published from the 1 April 2020 to the 8 May 2021 in the English language. The literature was searched on the following databases (PubMed Central, PubMed, Google Scholar, Cochrane database, science direct, and Ovid). The following keywords were used: “SARS-CoV-2” “COVID-19” “GBS” “Guillain–Barré syndrome”. MeSH: “Guillain–Barré syndrome AND COVID-19” “GBS AND COVID-19” “SARS-CoV-2 AND Guillain–Barré syndrome” “SARS-CoV-2 AND GBS”. The diagnostic criteria and classification used were published by the National Institute of Neurological Disorders (NINDS) and Stroke and Brighton Collaboration (Table 1) [9].

### Diagnostic Criteria

GBS is diagnosed mainly by clinical history and examination, and it is supported by some laboratory investigations, such as level of antiganglioside antibodies in the serum, CSF analysis, electrophysiological studies, and spinal cord and brain imaging. Magnetic resonance imaging (MRI) is helpful to rule out other differentials that occur acutely, such as infection, stroke, and space-occupying lesions. In contrast, normal values of these investigations do not exclude the diagnosis. Treatment is usually started even if the results are negative or delayed to improve the outcome and prevent complications [11].

## 3. Results

Based on the 105 cases in case reports and case series that we included in our review, we noticed that there were four types of GBS associated with the COVID-19 pandemic; according to the original articles, acute inflammatory demyelinating polyneuropathy (AIDP) was the most common type, and accounted for 33.33% of all case reports and series that we reviewed, while acute motor–sensory axonal neuropathy (AMSAN) accounted for 11.43%. Acute motor axonal neuropathy (AMAN) and Miller Fisher Syndrome (MFS) accounted for 8.57 and 6.67%, respectively. Finally, 38.18% of cases were unspecified. According to GBS types by NINDS and Brighton Collaboration, the following types have been delineated: classic sensorimotor GBS was most common type and accounted for 56.19% of all cases, pure motor GBS is the second most common type, accounted for 17.14% of cases, bilateral facial palsy with paresthesia accounted for 14.29% of cases, paraparesis accounted for 9.52% of cases, and MFS and bickerstaff brain stem encephalitis accounted for 7.62 and 2.86% of cases, respectively (Table 1).

According to demographic data, the average age of the patients was 56.6. The most common gender was male, accounting for 59.05% of cases. Overall, 29.52% of patients had known cases of hypertension, 12.38% of patients had a history of type two diabetes, and 18.10% of patients had no past medical history. The estimated average time between the emergence of COVID-19 symptoms and GBS symptoms was 15.77 days. Meanwhile, 58.10% of patients demonstrated postinfectious patterns and 35.24% displayed para-infectious patterns. Additionally, two of the reported patients were father and daughter, indicating a genetic predisposition (Table 2).

The most common COVID-19 symptoms were fever (55.24%) and cough (51.43%). Other symptoms included shortness of breath (27.62%), myalgia, and arthralgia (16.19%). Headache and hypo/dysgeusia were observed in 15.24% of patients. Meanwhile, 14.29% of patients presented with gastrointestinal symptoms and 12.38% of patients were reported as having hypo/anosmia. Generalized body ache and respiratory failure were present in 6.67% of patients. Odynophagia was reported in 5.71% of patients. Other less common symptoms were chest pain (4.76%), sore throat (3.81%), and chills and night sweats (2.86%). Rhinorrhea, sinonasal congestion, and confusion were reported in only 1.90% of patients. One patient was reported to have a rash (Table 3).

The neurological symptoms in COVID-19 patients with GBS were limb weakness (76.19%), paresthesia or pain (49.52%), and gait impairment (25.71%). Patients with cranial nerve symptoms accounted for 18.10%. Dysautonomic symptoms were reported in 15.24% of patients. Diplopia, which is a symptom of MFS, was reported in 5.71% of patients, and 2.86% of patients were reported to have bulbar symptoms. One patient was reported to have a dropping head (Table 4).

The most-reported clinical features of GBS were hypo/hyper/areflexia (88.57%), limb weakness (75.24%), sensory abnormality (51.43%), facial weakness (unilateral or bilateral) (29.52%), cranial nerve deficits (20.95%), and mechanical ventilation was used in 17.14% of patients. Cerebellar dysmetria and dysautonomic signs were observed in 15.24% and 12.38% of patients. Ophthalmoparesis was observed in 8.57% of patients, 9.52% of patients had bulbar weakness, and neck flexion weakness was observed in 4.76% of patients. Coarse resting tremor, fasciculations, truncal dysesthesia (neuropathy), and a positive Romberg test were reported in only 0.95% of patients (Table 5).

The serum abnormalities in patients included in the review are as follows: 41.90% of the patients had high inflammatory markers, 16.19% had leukocytosis, 15.24% had lymphocytopenia, and 11.43% had high D-dimers. Meanwhile, 7.62% of the patients had leukocytopenia, 5.71% had neutropenia, 5.71% had elevated liver enzymes, 4.76% had thrombocytopenia, 4.76% of the patients had hyperfibrinogenemia, 3.81% had hyponatremia, and 1.90% had high creatine phosphokinase (CPK). Finally, monocytosis and thrombocythemia were reported in 0.95% of patients (Table 6).

The serum antibody analysis showed that 9.95% of patients had SARS-Cov-2 IgG, 4.76% had high serum IgM, 3.81% had antiganglioside antibodies, and 1.90% had Mycoplasma pneumonia (MP) IgG. Human immunodeficiency virus (HIV) IgG, Cytomegalovirus (CMV) IgG, Epstein–Barr virus (EPV) IgG, and Herpes simplex virus (HSV) IgM/IgG were reported in only 0.95% of patients (Table 7).

MRI of the spine was normal in 15.24% of the patients, and it revealed degenerative changes abnormalities in 6.67%. Brain imaging was performed in less than half of the patients; it was normal in 26.67% of the patients, and it showed signs of demyelination in 2.86%. Chest imaging was normal in 10.48% of the patients, and it showed ground-glass opacities in both lungs in 18.10% of the patients (Table 8).

CSF analysis showed that 68.57% of the patients had albuminocytologic dissociation, 9.80% had an oligoclonal band, 8.57% had pleocytosis, and 5.71% had normal CSF. Oligoclonal band and high glucose were reported in 4.76% of patients. A high IgG index and SARS-CoV-2 (PCR) were reported in 3.80 and 1.90% of the patients, respectively. Nasal or throat swab SARS-CoV-2 PCR was positive in 80.00% and negative in 14.29% of the patients (Table 9).

Most of the patients, 59 out of 105 (56.19%), were treated with intravenous immunoglobulin (IVIG) at 0.4 g/kg daily for five days. IVIG and antiviral drugs were used in 20 (19.05%) patients. 

Thirteen patients were treated with five sessions of plasma exchange, and two of these received antiviral drugs. Six patients were treated with both plasma exchange and IVIG; one of them received antiviral drugs and two received IVIG after plasma exchange. Hydroxychloroquine was used in 33 patients (31.43%), 10 (9.52%) received methylprednisolone, six (5.71%) received dexamethasone, 15 (14.29%) received azithromycin, and seven (6.67%) patients received tocilizumab. A patient with MFS had mild symptoms and did not receive any treatment [12]. One patient had an excellent modified Erasmus GBS Outcome Score (mEGOS), and so did not receive treatment [13]. One more patient was symptomatically treated with acetaminophen and had a complete recovery [14]. One patient received a low dose of oral prednisone in addition to hydroxychloroquine and antiviral drugs [15].

Responses to treatment were reported in 99 out of 105 (94.29%) patients. Most of the patients showed improvement at four weeks duration (70.48%). Some patients improved at eight weeks. The majority of IVIG patients had a good outcome. 

The most significant complications were as follows: three patients developed hyponatremia, three patients reported depression, and two patients developed urinary retention. One patient developed heparin-induced thrombocytopenia, one patient had deep vein thrombosis and bacterial infection, one patient developed persistent urinary incontinence and transient fecal incontinence, constant neuropathic pain was seen in one patient, and one patient had a deficiency in zinc, folate, copper, and borderline B-12 [16,17,18,19,20,21,22,23].

After the administration of IVIG, one patient reported flushing and a pre-syncope episode, one progressively developed proximal weakness in all limbs, dysesthesia, and unilateral facial palsy, and another developed dysphagia with respiratory distress and hypoxia, requiring oxygen supplementation [17,24].

After the second session of plasma exchange, one patient had weakness that resulted in aspiration, and then a brief cardiac arrest required intubation and mechanical ventilation [24].

In the inpatient rehabilitation, one patient had resting tachycardia, persistent difficulty urination requiring catheterization, and features suggestive of MFS [25].

Eight patients died, two of them due to cardiac arrest, two developed multiple organ failure, one due to severe respiratory distress, one was hemodynamically unstable, one developed severe autonomic dysfunction, and the last one developed respiratory failure secondary to GBS [25,26,27,28,29,30,31].

## 4. Discussion

GBS is considered as one of the peripheral nervous system diseases usually present with lower motor neuron lesion signs: muscle atrophy, weakness, fasciculation, hypotonia, and hyporeflexia [32]. GBS is triggered through an anomalous autoimmune response to a prior infection against ganglioside components of the peripheral nerves (molecular mimicry), affecting various antigens in the demyelinating and axonal subtypes of GBS. Earlier, they discovered that coronavirus-type viruses (SARS and MERS) and Zika virus have been associated with GBS as well. Evidence indicates an association between COVID-19 and immune-mediated neurological complications such as GBS, but this is still unclear [33].

Through the process of analyzing the symptoms of GBS in COVID-19, some patients developed hyperreflexia instead of hyporeflexia, especially with the AMAN subtype. Other studies also describe the association between different GBS subtypes and hyperreflexia. Therefore, the presence of hyperreflexia should not delay the diagnosis and treatment of GBS. GBS patients with hyperreflexia have favorable prognosis than patients with hyporeflexia. For that reason, hyperreflexia must be included in the future diagnostic criteria of GBS [32].

The presence of IgG antibodies against SARS-CoV-2 supports the diagnosis of post-COVID-19 GBS. Additionally, the clinical features of post-COVID-19 GBS did not vary from individuals of causes linked to other viruses, with the significant exception of a tremendous respiratory involvement [5].

Antibodies for various gangliosides, such as GM1, GD1a, GT1a, GM2, and GQ1b, have been found in a small percentage of GBS patients, and their disappearance after clinical improvement suggests a pathogenetic role in the neuropathy. Antibodies to these proteins have been linked to a variety of clinical and electrophysiological characteristics of GBS. Anti-GM1 and anti-GD1a antibodies have been linked to an antecedent *Campylobacter jejuni* (*CJ*) infection which results in widespread motor and axonal impairment and a poor prognosis. Anti-GM2 antibodies to an antecedent *CMV* infection result in severe sensory–motor impairment, frequent respiratory impairment, and demyelinating features [34].

Not all patients were tested for antiganglioside antibodies, but most of the tested patients did not have antigangliosides antibodies, except four patients. Kajumba et al. suggested that the presence of antibodies is consistent with the theory of immune-mediated mechanisms of GBS, and the improvement of patients after immunoglobulin therapy supports that [35]. However, the absence of antiganglioside antibodies suggests non-immunologic mechanisms such as direct infection of the nervous system [35].

CSF results can change throughout a patient’s illness [36]. It was previously reported that a positive CSF PCR is highest when CSF is obtained between 3 and 14 days after the onset of neurological symptoms [37].

The results show that only two patients had positive SARS-CoV-2 PCR in the CSF; possible explanations include the absence of disruption in the blood–brain barrier (BBB) that allows SARS-CoV-2 to cross the CSF space [38] and the low sensitivity of currently available rRT-PCR, at about 60% [39,40].

A cluster of eight Italian patients presented with GBS symptoms during the peak of SARS-CoV2 infection [41]. These cases prove the genetic factor’s role in the development of GBS in COVID-19 patients. 

In almost all cases, intravenous immunoglobulins were used. IVIG and plasma exchange have the same efficacy [11]. Most patients had a favorable outcome.

GBS is usually a postinfectious syndrome, as demarcated by an onset that is later than the acute symptoms of infection and by a mechanism that is distinct from the infection [33]. The most identified precipitants are HIV, HSV, CMV, EPV, MP, Cj, Varicella-zoster virus (VZV), Influenza-A virus, and Haemophilus influenza [42]. 

Some cases and reports discussed the effect of SARS and MERS infection on the nervous system and GBS development and confirmed that patients might develop acute polyneuropathy. After the onset of SARS-CoV-2 by 21 days and according to one of the case reports, three patients showed neuropathy, myopathy, and peripheral nerve disorders. On the other hand, acute polyneuropathy is the common type of GBS in patients with MERS-CoV infection [9].

The association between COVID-19 and GBS has recently been described. It takes the form of a para-infectious profile as an alternative to the usual postinfectious profile. Para-infectious neuropathies could progress as an unusual hyperimmune response, and they could also represent a direct toxic or neuropathic effect. It has been described as a potential uncommon sequela of COVID-19 infection through the first reported case in Wuhan, which suggested a para infectious presentation. Evidence suggests multiple mechanisms behind it; the mechanism strongly associated with COVID-19 and immune-mediated neurological complication is molecular mimicry between SARS-CoV-2 and human autoantigens. It was found that there are two SARS-CoV-2 hexapeptides that mimic the human shock protein (HSP 90B, 90B2, and 60I). High levels of autoantibodies against different familial HSP were found in the CSF and serum of those patients [33].

Subtypes of GBS have different pathogenesis. In the AMAN subtype of GBS, the infecting organisms possibly share similar epitopes to a component of the peripheral nerves (molecular mimicry); therefore, the immune responses crossreact with the nerves, causing axonal degeneration. Patients with AMAN commonly have serum antibodies against GM1, GM1b, GD1a, and GalNAc-GD1a gangliosides. In the AIDP type, the immune system reacts against target epitopes in Schwann cells or myelin result in demyelination; however, the exact target molecules in the case of AIDP have not yet been identified. Patients with MFS commonly have antibodies against GD1b, GD3, GT1a, and GQ1b gangliosides, which are related to ataxia and ophthalmoplegia [4].

The scale of COVID-19 infection ranges from asymptomatic to serious infection, and varies from other viral pulmonary infections, which require particular care as they could be dominated by the severity of pulmonary and cardiac symptoms [26]. This implies that COVID-19 pneumonia may overlap with GBS-associated respiratory muscle weakness, increasing the number of cases requiring respiratory assistance [5]. The alarming respiratory markers indicate an overlap between SARS-CoV-2 pneumonia and a neuromuscular condition involving respiratory muscle such as GBS. Initially, the diagnosis of GBS needs to be assumed in patients with COVID-19 who develop symptoms of diaphragmatic weakness (basal atelectasis on chest x-ray, development of hypercapnia in arterial blood gas analysis). Next, in patients with supposed or diagnosed acute neuromuscular disease in the sequence of COVID-19 infection, the effective monitoring of the respiratory muscles is suggested additionally to a careful neurological assessment, and it is serious in determining the timing of intubation regardless of the degree of respiratory failure from SARS-CoV-2 infection. In intensive care unit (ICU) patients with COVID-19 infection, the development of diaphragmatic weakness is not rare; consequently, a delicate differential diagnosis between ICU-acquired weakness and GBS must be taken into consideration, as the two situations need different curative approaches [26].

Over time, variants of the SARS-CoV-2 virus were identified: Alpha, Beta, Gamma, and Delta. Variants produce the same symptoms of COVID-19 but spread faster than other variants of SARS-CoV-2, and their severity is different from each other’s. Beta and Gamma cannot cause severe illness or death. In contrast, Alpha and Delta may cause severe illness or death. All vaccines are considered effective against these variants. The effectiveness of vaccines may change against new variants that could arise in the future [43].

Our review had some strengths. Firstly, the inclusion of 73 cases report [5,12,13,15,16,17,18,19,20,21,22,23,27,29,30,31,44,45,46,47,48,49,50,51,52,53,54,55,56,57,58,59,60,61,62,63,64,65,66,67,68,69,70,71,72,73,74,75,76,77,78,79,80,81,82,83,84,85,86,87,88,89,90,91,92,93,94,95,96,97,98,99,100,101], and 10 case series [14,25,28,102,103,104,105,106,107,108]. Secondly, confirmed COVID-19 by PCR. Moreover, GBS was confirmed by clinical, laboratory investigations, CSF analysis, and electrophysiological studies. Thirdly, the reported comorbidities are not known to cause GBS. 

One of the limitations is that we included only cases published in English. As both diseases affect the respiratory system, some COVID-19 symptoms could be attributed to GBS and vice versa. 

## 5. Conclusions

The association between COVID-19 and GBS is unclear, but there is one mechanism strongly associated with COVID-19 and immune-mediated neurological complications, which is the molecular mimicry between SARS-coV-2 and human autoantigens. The most common subtype is a classic sensorimotor, which accounted for 56.19% of all cases. It is crucial to assume GBS in COVID-19 patients who developed diaphragmatic weakness. Our review signifies the association between COVID-19 and GBS; however, further studies are needed to confirm it. 

## Figures and Tables

**Table 1:** Subtypes of GBS.

Subtype by Original Article	Number (%)	Subtype by NINDS and Brighton Collaboration	Number (%)
(AMSAN)	12 (11.43)	Classic sensorimotor GBS	59 (56.19)
(AIDP)	35 (33.33)	Pure motor	18 (17.14)
(MFS)	7 (6.67)	Paraparetic	10 (9.52)
(AMAN)	9 (8.57)	Pharyngeal-cervical brachial	2 (1.90)
unspecified	40 (38.10)	Bilateral facial palsy with paresthesia	15 (14.29)
		Pure sensory	0 (0.00)
		Miller Fisher syndrome	8 (7.62)
		Bickerstaff brainstem encephalitis	3 (2.86)

**Table 2:** Demographic data.

Age	Number (%)
10–19	3 (2.86)
20–29	4 (3.81)
30–39	11 (10.48)
40–49	10 (9.52)
50–59	29 (27.62)
60–69	26 (24.76)
70–79	17 (16.19)
80–89	4 (3.81)
90–99	1 (0.95)
**Gender**	**Number (%)**
Male	62 (59.05)
Female	43 (40.95)
**Ethnicity**	**Number (%)**
Georgian	1 (0.95)
Italian	1 (0.95)
Moroccan	1 (0.95)
South Asian	1 (0.95)
Iranian	2 (1.90)
Guinea	1 (0.95)
Caucasian	7 (6.67)
Indian	1 (0.95)
Hispanic	3 (2.86)
**Past medical history**	**Number (%)**
No past history	19 (18.10)
Not mentioned	30 (28.57)
Type 2 diabetes	13 (12.38)
Hypertension	31 (29.52)
Hyperlipidemia	6 (5.71)
Coronary artery disease	3 (2.86)
Heart rhythm disease	1 (0.95)
Hypothyroidism	3 (2.86)
Breast cancer	5 (4.76)
Rheumatoid arthritis	3 (2.86)
Rest leg syndrome	1 (0.95)
Chronic back pain	2 (1.90)
Psoriasis	1 (0.95)
Obesity	6 (5.71)
Reflex sympathetic dystrophy	1 (0.95)
Fibromyalgia	2 (1.90)
Asthma	4 (3.81)
Hiatal hernia	1 (0.95)
Cholelithiasis	1 (0.95)
Benign prostatic hyperplasia	1 (0.95)
Abdominal aortic aneurysm	2 (1.90)
Chronic kidney disease	2 (1.90)
Chronic myelogenous leukemia	1 (0.95)
Cervical spondylosis and disc herniation	1 (0.95)
Lumper stenosis	1 (0.95)
Gout	1 (0.95)
Osteoporosis	2 (1.90)
Major depressive disorder	2 (1.90)
Trigeminal neuralgia	1 (0.95)
Chronic obstructive pulmonary disease	1 (0.95)
Migraine	1 (0.95)
Stroke	1 (0.95)
**Days between COVID-19 symptoms and GBS symptoms**	**Number (%)**
0 day	16 (15.24)
1 day	4 (3.81)
3 days	1 (0.95)
4 days	1 (0.95)
5 days	6 (5.71)
6 days	1 (0.95)
7 days	8 (7.62)
10 days	13 (12.38)
12 days	2 (1.90)
14 days	14 (13.33)
16 days	3 (2.86)
17 days	2 (1.90)
21 days	15 (14.29)
30 days	6 (5.71)
60 days	3 (2.86)
90 days	2 (1.90)
210 days	1 (0.95)
Postinfectious	61 (58.10)
Para-infectious	37 (35.24)
	**Number (%)**
Genetic history	3 (2.86)

**Table 3:** COVID-19 symptoms.

COVID-19 Symptom	Number (%)
Fever	58 (55.24)
Dry or wet cough	54 (51.43)
Shortness of breath	29 (27.62)
Myalgia and arthralgia	17 (16.19)
Headache	16 (15.24)
Hypogeusia or dysgeusia	16 (15.24)
Gastrointestinal symptoms	15 (14.29)
Hyposmia or anosmia	13 (12.38)
Generalized body ache	7 (6.67)
Respiratory failure	7 (6.67)
Odynophagia	6 (5.71)
Chest pain	5 (4.76)
Sore throat	4 (3.81)
Chills and night sweats	3 (2.86)
Rhinorrhea	2 (1.90)
Sinonasal cognition	2 (1.90)
Confusion	2 (1.90)
Rash	1 (0.95)

**Table 4:** Neurological symptoms.

Neurological Symptom	Number (%)
Limb weakness	80 (76.19)
Paresthesia or pain	52 (49.52)
Gait impairment	27 (25.71)
Cranial nerve symptoms	19 (18.10)
Dysautonomic symptoms	16 (15.24)
Diplopia	6 (5.71)
Bulbar symptoms	3 (2.86)
Dropping head	1 (0.95)

**Table 5:** Neurological examination findings.

Neurological Examination Finding	Number (%)
Hyperreflexia, hyporeflexia or areflexia	93 (88.75)
Limb weakness	79 (75.24)
Sensory abnormality	54 (51.43)
Unilateral or bilateral facial weakness	31 (29.52)
Cranial nerve deficits	22 (20.95)
Mechanical ventilation	18 (17.14)
Cerebellar dysmetria	16 (15.24)
Dysautonomic signs	13 (12.38)
Bulbar weakness	10 (9.52)
Ophthalmoparesis	9 (8.57)
Neck flexion weakness	5 (4.76)
Truncal dysesthesia	1 (0.95)
Fasciculation	1 (0.95)
Coarse resting tremor	1 (0.95)
Romberg test positive	1 (0.95)

**Table 6:** Serum abnormality analysis.

Serum Abnormality Analysis	Number (%)
High inflammatory markers	44 (41.90)
Leukocytosis	17 (16.19)
Lymphocytopenia	16 (15.24)
High D-dimers	12 (11.43)
High liver enzyme (GGT, AST, ALT)	6 (5.71)
Thrombocytopenia	5 (4.76)
Hyperfibrinogenemia	5 (4.76)
Hyponatremia	4 (3.81)
Neutropenia	2 (5.71)
High CPK	2 (1.90)
High monocytes	1 (0.95)
Thrombocythemia	1 (0.95)

**Table 7:** Serum antibody analysis.

Serum Antibody	Number (%)
SARS-CoV-2 IgG	10 (9.52)
High serum IgM	5 (4.76)
Antigangliosides antibodies	4 (3.81)
MP IgG	2 (1.90)
HIV IgG	1 (0.95)
EBV IgG	1 (0.95)
CMV IgG	1 (0.95)
HSV IgM/IgG	1 (0.95)

**Table 8:** MRI and CT imaging.

Spine	Number (%)
Normal	16 (15.24)
Degenerative changes in the spine	7 (6.67)
Lumbosacral root enhancement	3 (2.86)
Abnormal enhancement in the cauda equina	3 (2.86)
T2-hypersensitivity	2 (1.90)
Asymmetrical thickening and hyperintensity of post-ganglionic roots supplying the brachial and lumbar plexuses in short-tau inversion	1 (0.95)
Brainstem and cervical meningeal enhancement	1 (0.95)
Intervertebral disc herniation	1 (0.95)
**Brain**	**Number (%)**
Normal	28 (26.67)
Signs of demyelination	3 (2.86)
Facial (VII), abducens (VI) nerves bilaterally, and the right oculomotor nerve (III) enhancement	1 (0.95)
Oculomotor, facial, and vestibulocochlear cranial nerves Enhancement	1 (0.95)
Evidence of polyneuropathy	1 (0.95)
Acute infarct in the left centrum semiovale	1 (0.95)
Mild small vessel ischemic disease of the white matter, with no Significant focal lesions	1 (0.95)
Subcortical lesions	1 (0.95)
Chronic microvascular ischemic changes	1 (0.95)
**Chest imaging**	**Number (%)**
Ground glass opacities in both lungs	19 (18.10)
Normal	11 (10.48)
Bilateral basilar opacities	6 (5.71)
Bilateral ill-defined infiltrates	3 (2.86)
Diffuse consolidation and pleural effusion	2 (1.90)
Mild bilateral patchy high-density shadows	2 (1.90)
Bilateral lower lobe consolidations with air bronchograms	1 (0.95)
Bilateral pulmonary consolidation is more prominent in the periphery of the lung bases	1 (0.95)
Bilateral basilar atelectasis and small pleural effusions	1 (0.95)
Bilateral interstitial pneumonia	1 (0.95)
Interstitial and alveolar pattern with both lungs	1 (0.95)

**Table 9:** Cerebrospinal fluid analysis and SARS-CoV-2 PCR.

Cerebrospinal Fluid	Number (%)
Albuminocytologic Dissociation	72 (68.57)
Pleocytosis	9 (8.57)
Normal	6 (5.71)
High glucose	5 (4.76)
Oligoclonal band	5 (4.76)
High IgG index	4 (3.80)
SARS-CoV-2 PCR	2 (1.90)
**Nasal or throat swab SARS-CoV-2 PCR**	**Number (%)**
Positive	84 (80.00)
Negative	15 (14.29)
